# Ondansetron Reduces the Incidence of Hypotension after Spinal Anaesthesia: A Systematic Review and Meta-Analysis

**DOI:** 10.3390/ph15121588

**Published:** 2022-12-19

**Authors:** Xiao-Min Hou, Yan-Jun Chen, Lan Lai, Ke Liu, Qi-Hong Shen

**Affiliations:** Department of Anesthesiology, Affiliated Hospital of Jiaxing University, Jiaxing 314000, China

**Keywords:** ondansetron, hypotension, meta-analysis, spinal anaesthesia

## Abstract

Hypotension induced by spinal anaesthesia is a common clinical complication associated with multiple perioperative adverse events. We conducted a systemic review and meta-analysis to confirm whether ondansetron could alleviate hypotension following spinal anaesthesia. PubMed, Embase, Web of Science, and Cochrane Library were searched to identify eligible randomised controlled trials from their respective database inception dates to 30 September 2022. The primary outcome of the meta-analysis was the incidence of hypotension after spinal anaesthesia. The risk of bias in the included studies was evaluated using the revised Cochrane risk of bias tool for randomised trials (RoB 2.0). Grading of Recommendations, Assessment, Development, and Evaluation was applied to assess the level of certainty. A total of 25 studies were included in this research. The meta-analysis revealed that ondansetron significantly decreased the incidence of hypotension (RR = 0.65, 95% CI 0.53–0.80, *p* < 0.01, *I*^2^ = 64%) and bradycardia. In addition, patients treated with ondansetron had a reduced need for vasopressors administration. This study suggests that ondansetron may be recommended as a prophylaxis for hypotension and bradycardia following spinal anaesthesia; the level of evidence was moderate with a high level of heterogeneity.

## 1. Introduction

The recommended anaesthetic technique for various surgical procedures, including caesarean section, orthopaedic surgery, and lower abdominal general surgery, is spinal anaesthesia, also known as a subarachnoid block. However, hypotension caused by spinal anaesthesia is a common problem that plagues doctors and patients, occurring in approximately 80% of cases [1]. A reduction in blood pressure can cause a series of intraoperative adverse effects, such as dizziness, nausea and vomiting, regurgitation, and aspiration [2]. Therefore, it is important to investigate methods to reduce the incidence of hypotension following spinal anaesthesia that are economical, safe, and effective.

The mechanism of hypotension following spinal anaesthesia involves the reduction in vascular resistance caused by the sympathetic block and the activation of the Bezold–Jarisch reflex, leading to vasodilation and hypotension [3]. Peripheral serotonin receptors, 5-Hydroxytryptamine3 (5HT3), are required for the activation of the Bezold–Jarisch reflex [4]. In a rabbit model, a 5-HT3 receptor antagonist was reported to suppress bradycardia and hypotension by preventing the Bezold–Jarisch reflex [5]. Thus, numerous clinical trials have been performed to demonstrate the preventive effect of ondansetron on hypotension following spinal anaesthesia [6,7,8]. A meta-analysis published in 2016 argued that 5HT3 receptor antagonists effectively reduced the incidence of spinal anaesthesia-induced hypotension in patients undergoing caesarean sections but had no significant effect on a nonobstetric population [9]. A recent meta-analysis that was focused on the nonobstetric population treated with ondansetron contradicted that viewpoint [10]. However, in the aforementioned studies, the sample sizes were relatively small. Furthermore, significant resources have been utilised annually on clinical trials on this topic worldwide [6,7,11,12,13,14]. Therefore, we conducted this systemic review and meta-analysis using trial sequential analysis (TSA) to investigate whether ondansetron prevents hypotension following spinal anaesthesia.

## 2. Methods

This systematic review and meta-analysis were performed and reported according to the recommendations of the preferred reporting items for systematic reviews and metanalyses. The registration number of the International Prospective Register of Systematic Reviews (PROSPERO) was CRD 42022353540.

### 2.1. Systematic Literature Search

The electronic databases of Web of Science, PubMed, Cochrane Library, and Embase were systematically searched. The retrieval date was from database establishment to 30 September 2022 without language limitations. The search terms included the following: “ondansetron”, “5-HT3 receptor antagonists”, “5-Hydroxytryptamine3”, “spinal anaesthesia”, “intrathecal anaesthesia”, and “subarachnoid anaesthesia”. Furthermore, the references of the eligible studies were also searched systematically.

### 2.2. Criteria for Selection

The eligibility requirements for inclusion were as follows: (1) Participants (P): patients receiving spinal anaesthesia. (2) Intervention (I): trials reporting ondansetron was administered intravenously. (3) Comparison (C): placebo. (4) Outcome (O): trials reporting the incidence of hypotension was one of the outcomes. (5) Study designs (S): randomised controlled trials (RCTs).

The following were the exclusion criteria for this research: (1) Combined with other types of anaesthesia, such as epidural and general anaesthesia. (2) Animal studies. (3) Incomplete studies, such as conference abstracts. (4) Ondansetron was administered by other means.

### 2.3. Extraction of Data and Outcomes

First, EndNote was used independently by two authors to exclude the duplicates. Second, they determined whether the trials met the conditions according to the title and abstract. Finally, the full texts of the screened studies were then carefully examined to determine whether they met all the inclusion criteria. Using data from the included studies, the two authors independently retrieved and cross-checked the following information: the author’s name, year of publication, type of surgery, sample size, patients, blinding methods, details of spinal anaesthesia, dosage and timing of ondansetron, and the definition of hypotension. We emailed the corresponding authors of the research where some information was unavailable in the published articles. We sent another email enquiry if there was no response after more than a week.

The primary outcome of this study was the incidence of hypotension following spinal anaesthesia. (The definition of hypotension was based on that used in each clinical study) Secondary outcomes included the incidence of bradycardia, the use of vasopressor administration, and the dosage of ephedrine. If different doses or different types of ondansetron were studied, we combined the dichotomous variables for the meta-analysis. In the case of continuous variables, we analysed the data for different groups.

### 2.4. Evaluation of the Quality and the Risk of Bias

A revised Cochrane risk of bias tool for randomised trials (RoB 2.0) was used to assess the risk of bias in the included studies. The risk of bias table included bias from the process of randomisation, bias due to deviations from the expected interventions, bias from missing data, bias from the measurement of the outcome, and bias from the selection of the reported results. Each trial was assessed as either high risk, some concerns, or low risk. 

The degree of confidence was assessed utilizing the Grading of Recommendations, Assessment, Development, and Evaluation (GRADE). Accordingly, the level of certainty was categorised as very low, low, moderate, or high.

### 2.5. Statistical Analysis

The meta-analysis was performed using the Review Manager 5.3 (version 5.3, Copenhagen, Denmark) statistical software and Stata version 12.0 (Stata Corp LP, College Station, TX, USA). The pooled risk ratio (RR) and 95% confidence intervals (CIs) for dichotomous outcomes were calculated. For continuous data, the mean differences (MD) and 95% CIs were evaluated. Statistical significance was considered to be achieved when the *p*-value was <0.05. The number needed to treat (NNT) was calculated for statistically significant outcomes. The heterogeneity in the trials was examined utilizing the *I*^2^ statistic, wherein *I*^2^ > 50% was defined as “highly heterogeneous”. Clinical and methodological issues were shown to be the primary causes for high clinical heterogeneity. Consequently, a random-effects model was utilised even in studies with low *I*^2^ values.

Subgroup analyses were performed according to the different dosages of ondansetron (≤4 mg vs. >4 mg) and type of surgeries (caesarean section vs. non-caesarean section). For the trials that did not report the type of surgery and clearly did not belong to caesarean section (e.g., only included elderly patients, men, or specifically non-obstetric surgery), we analysed them in the non-caesarean section group. Funnel plots and Egger’s test were employed to assess the publication bias. In addition, a sensitivity analysis was performed to test the stability of the primary outcome.

Using TSA software (version 0.9.5.10 beta), we performed a TSA method to control the risk of type I error caused by repeated testing. When the cumulative z-curve crosses the TSA monitoring boundary or enters the required information size line, no further study is required [15]. The risk of type 1 error was set as 5% with two-sided, and the power was 80%.

## 3. Results and Discussion

### 3.1. Search Results

**Figure 1:**
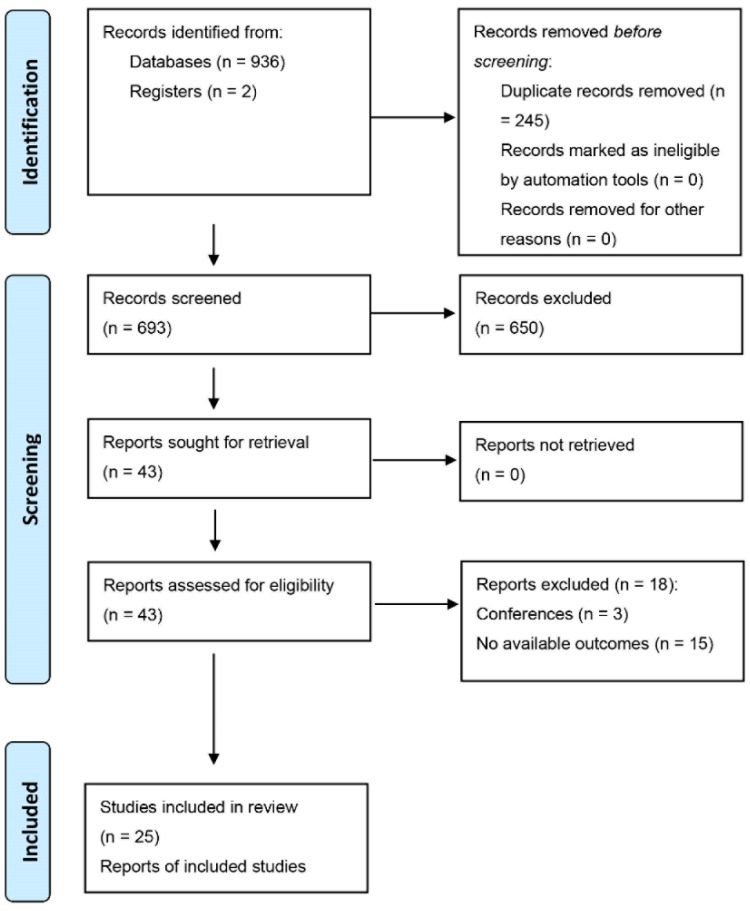
The literature retrieval and screening process according to the PRISMA guidelines.

According to the retrieval strategy, a total of 938 related studies were initially obtained from the databases. First, 245 duplicates were excluded, following which, 650 studies were removed once their titles and abstracts were reviewed. To determine whether the remaining 43 studies met the criteria for inclusion, their full texts were carefully analysed. Notably, 18 additional trials were omitted for the following reasons: conference abstracts (*n* = 3) [16,17,18] and no available outcomes (*n* = 15) [6,8,14,19,20,21,22,23,24,25,26,27,28,29]. Finally, a total of 25 trials that satisfied the eligibility requirements were selected for inclusion in the meta-analysis [2,11,12,13,30,31,32,33,34,35,36,37,38,39,40,41,42,43,44,45,46,47,48,49,50]. The schematic of the process of literature screening is depicted in Figure 1.

### 3.2. Study Characteristics

In total, 25 RCTs comprising 2536 patients (1405 patients in the ondansetron group and 1131 patients in the control group) were analysed. The publication years for these studies were from 2005 to 2021, and the sizes of the samples were within a range of 40–254. The dosage of ondansetron ranged from 2 mg to 12 mg. One study was investigator-blinded [42], another study was patient-blinded [34], and the remaining studies were double-blinded. Only one trial did not clearly define hypotension following spinal anaesthesia [45]. Table 1 provides the detailed data on the specific features of the included studies.

### 3.3. Assessment of Bias

**Figure 2:**
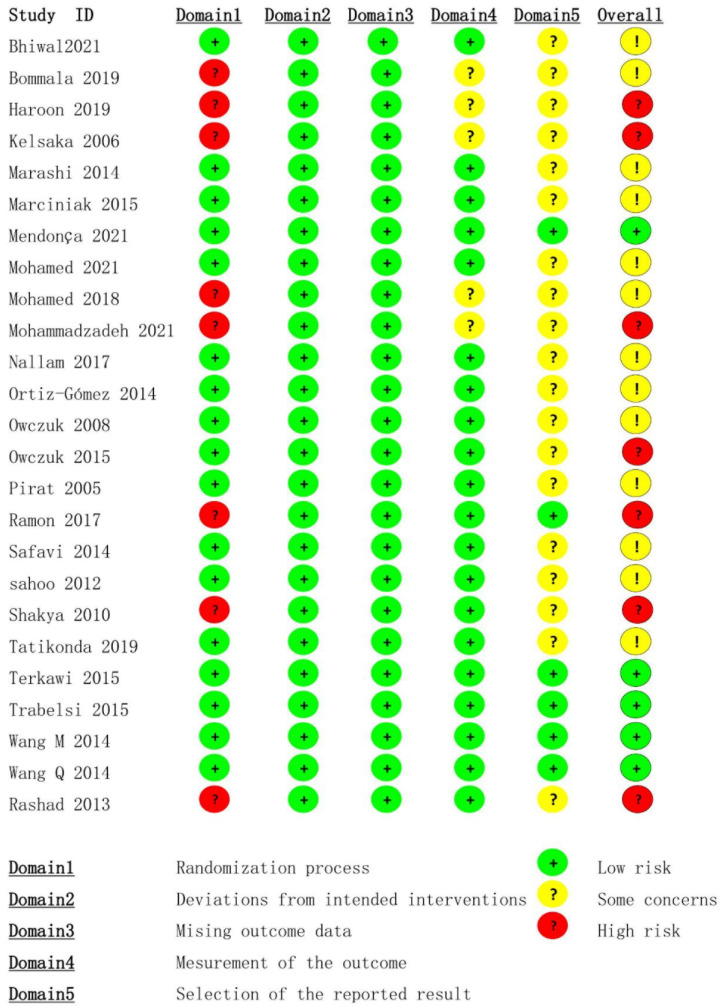
Summary of the risk of bias results for the included studies [2,11,12,13,30,31,32,33,34,35,36,37,38,39,40,41,42,43,44,45,46,47,48,49,50].

Eight trials had a high risk of bias in the “randomisation process”, while five trials had some concerns of bias in the “measurement of the outcome”. Only six trials had a pre-registered protocol, and the “selection of the reported results” was graded as low risk. Figure 2 presents the overall findings of the bias evaluation.

### 3.4. The Incidence of Hypotension

**Figure 3:**
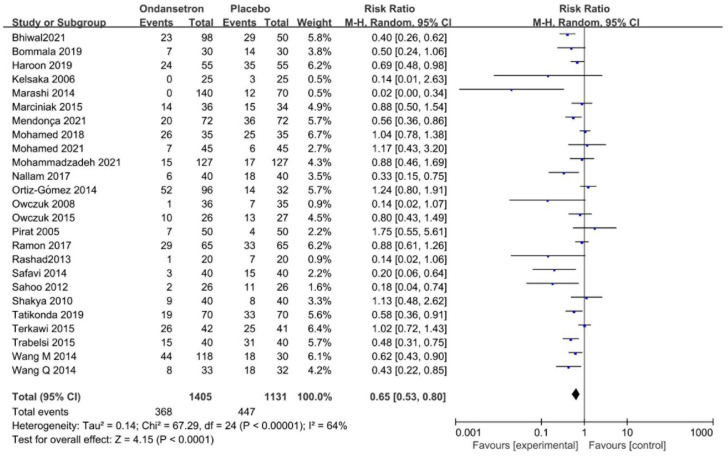
Forest plot of the pooled analysis showing the incidence of hypotension [2,11,12,13,30,31,32,33,34,35,36,37,38,39,40,41,42,43,44,45,46,47,48,49,50].

**All the included trials reported the incidence of hypotension.** The meta-analysis demonstrated that ondansetron reduces the occurrence of hypotension as compared to the control group, with high heterogeneity (RR = 0.65, 95% CI 0.53–0.80, *p* < 0.01, *I*^2^ = 64%, Figure 3). The NNT was 7.5. Subgroup analyses were performed to explore the sources of heterogeneity according to different dosages of ondansetron and surgical modalities. However, heterogeneity was not significantly reduced (Supplementary Figures S1 and S2).

### 3.5. The Incidence of Bradycardia

**Figure 4:**
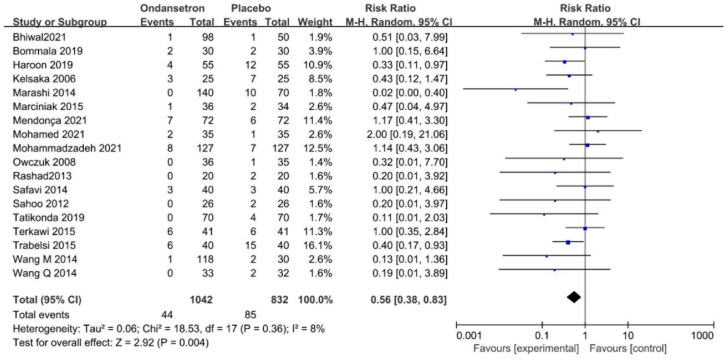
Forest plot of the pooled analysis showing the incidence of bradycardia [2,11,12,13,30,31,32,33,35,38,42,43,44,46,47,48,49,50].

Eighteen trials recorded the incidence of bradycardia. The forest plot demonstrated that patients treated with ondansetron had a significantly lower occurrence of bradycardia, with low heterogeneity (RR = 0.56, 95% CI 0.38–0.83, *p* < 0.01, *I*^2^ = 8%, Figure 4). The NNT was 16.7.

### 3.6. Rescue of Vasopressor Administration

**Figure 5:**
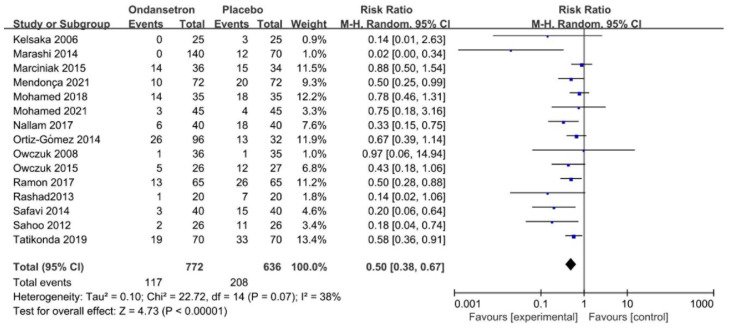
Forest plot of the pooled analysis showing the number of patients who required vasopressor [2,11,12,32,33,34,36,37,38,39,41,42,43,44,46].

Fifteen trials assessed the number of patients who required vasopressor administration. The forest plot indicated that ondansetron significantly reduced the number of patients who required vasopressor administration following spinal anaesthesia, with low heterogeneity (RR = 0.50, 95% CI 0.38–0.67, *p* < 0.01, *I*^2^ = 38%, Figure 5).

**Figure 6:**
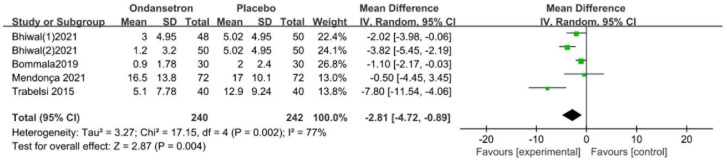
Forest plot of the pooled analysis showing the dose of administered ephedrine [12,13,30,48].

Four trials reported the dosage of administered ephedrine following spinal anaesthesia. The result revealed that patients in the ondansetron group had a lower dose of administered ephedrine, with high heterogeneity (MD = −2.81 mg, 95% CI [−4.72, −0.89], *p* < 0.05, *I*^2^ = 77%, Figure 6).

### 3.7. Publication Bias and Sensitivity Analysis

**Figure 7:**
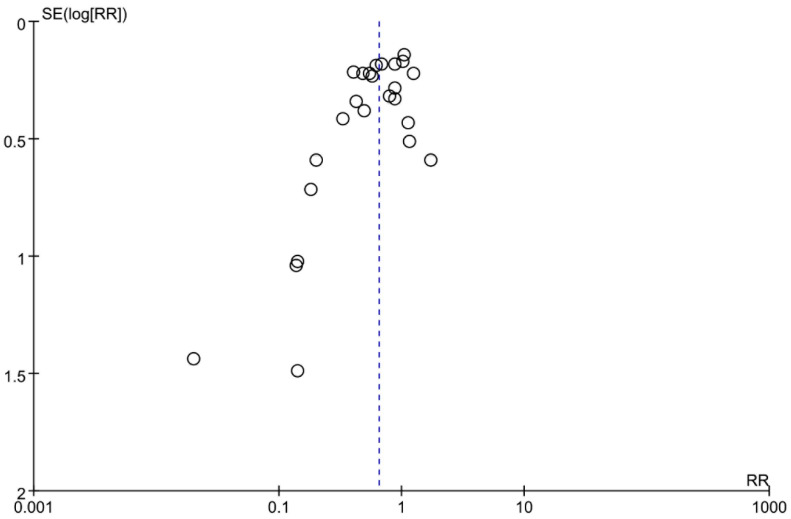
The funnel plot of the incidence of hypotension.

The funnel plot of the incidence of hypotension revealed a basically symmetric distribution, and the Egger’s test *p*-value was 0.554 (>0.05), suggesting there was no obvious publication bias (Figure 7). Sensitivity analysis was performed on the incidence of hypotension with unchanged effect estimates, indicating the robustness of the pooled result (Supplementary Figure S3).

### 3.8. Trial Sequential Analysis

**Figure 8:**
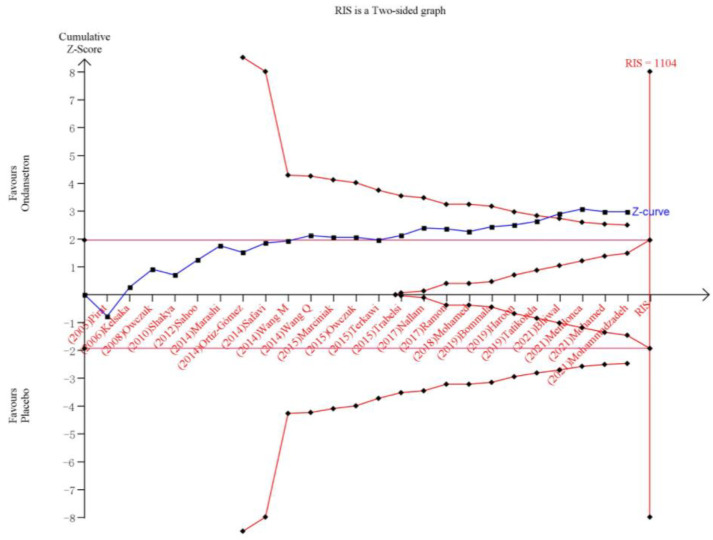
The trial sequential analysis result for the incidence of hypotension.

The TSA result for hypotension incidence showed that the cumulative z-curve had reached both the traditional and TSA boundaries, and that further studies were not required to confirm this evidence (Figure 8).

### 3.9. Grade Evaluation

All the studies considered in this review used the randomised trial “study design” type. The *I^2^* values of some reports were high to a relative extent, while the “inconsistency” was graded as serious. No obvious publication bias was evaluated among the evidence. The overall GRADE results are summarised in Table 2.

### 3.10. Discussion

This systematic analysis and meta-analysis revealed that the prophylactic administration of ondansetron may significantly reduce the risks of hypotension (moderate-quality evidence), bradycardia (high-quality evidence), and the need for vasopressor administration rescue (high-quality evidence) in patients undergoing spinal anaesthesia. According to the TSA, there was sufficient evidence to support the fact that ondansetron prevents hypotension after spinal anaesthesia.

In recent years, studies have been conducted on different treatments for hypotension following spinal anaesthesia, such as fluid therapy and vasopressors [51,52]. However, the activation of the Bezold–Jarisch reflex is one of the important mechanisms of hypotension after spinal anaesthesia. Numerous studies have focused on preventing hypotension by attenuating the Bezold–Jarisch reflex with 5-HT3 receptor antagonists [2,11,12,13,30,39,46,47,49,53]. Ondansetron was demonstrated to be effective in preventing hypotension in previous meta-analyses [9,10,54,55]. However, the promotion of its clinical application was constrained by the specific type of surgery and the limited sample size. Our meta-analysis of 25 RCTs revealed that ondansetron can significantly reduce the risks of hypotension (NNT 7.5) and bradycardia (NNT 16.7), which is consistent with previous studies. Sensitivity analysis results supported the robustness of the combined results of this study.

Notably, the TSA findings indicated that the information size for supporting the role ondansetron plays was sufficient, meaning that there is no need to spend more resources on clinical trials to investigate its effectiveness. In addition, we evaluated the preventive effect of ondansetron on hypotension by subgroup analysis at different doses (low-dose vs. high-dose) and different surgical types (caesarean section vs. non-caesarean section). The results of the subgroup analyses were consistent with the overall result. However, we did not conduct a meta-analysis for other types of 5-HT3 receptor antagonists due to the insufficient trials. Therefore, to extend this finding to other types of 5-HT3 receptor antagonists, additional high-quality RCTs are required.

In addition, we found that patients treated with ondansetron had a lower need for vasopressor administration and a lower dosage of administered ephedrine, which were consistent with previous studies [9,12]. This was mainly attributed to the lower incidence of hypotension. However, we did not conduct a meta-analysis for the dosage of phenylephrine due to the low number of trials. Recent studies have indicated that phenylephrine has become the first-line treatment because of a more favourable effect on neonatal pH compared with ephedrine [56].

There was significant clinical heterogeneity, which may be related to the different patient populations (age, disease, obstetric, sex, etc.), types of surgery, different dosages of ondansetron, type of spinal anaesthesia and drug, definition of hypotension, and the technique to measure blood pressure. Therefore, a random-effects model was adopted in this meta-analysis. Due to the high level of heterogeneity, the quality of evidence for the primary outcome was moderate.

This study had some limitations. First, some included studies did not clearly report the method of randomisation, leading to a decline in study quality. Second, meta-analyses for other types of 5-HT3 receptor antagonists were not conducted due to insufficient data. Third, some included studies did not report the type of surgery, which could lead to potential bias. Finally, definitions of hypotension and bradycardia varied across studies, causing potential bias.

## 4. Conclusions

This study provided evidence that ondansetron may serve as a clinical option for the prevention of hypotension and bradycardia following spinal anaesthesia. Further research is needed to determine whether the effect is similar for other types of 5-HT3 receptor antagonists.

## Figures and Tables

**Table 1:** The details of the included studies.

Study	Sample Size	Type of Surgery	Patients	Blinded Method	Spinal Anaesthesia	Dosage of Ondansetron	Definition of Hypotension
Bhiwal 2021 [13]	O (4 mg): 48	Caesarean section	ASA: I–II	Double-blinded	Position: left lateral	Ondansetron 4 mg or 8 mg before spinal anaesthesia	Reduction in SBP by > 20% from the baseline value.
	O (8 mg): 50		Age range: 18–40		Local anaesthetic: 2 mL 0.5% hyperbaric bupivacaine at L 3–4 or L 4–5		
	Control: 50						
Bommala 2019 [30]	O (4 mg): 30	Nonobstetric surgery	ASA: I–II	Double-blinded	Position: lateral decubitus	Ondansetron 4 mg before spinal anaesthesia	Reduction in SBP by > 30% from the baseline value or SBP < 90 mmHg.
	Control: 30		Age range: 18–60		Local anaesthetic: 3 mL 0.5% hyperbaric bupivacaine at L 3–4		
Haroon 2019 [31]	O (9 mg): 55	NR	ASA: I–III	Double-blinded	Position: Sitting	Ondansetron 9 mg before spinal anaesthesia	Reduction in SBP by > 21% from the baseline value or SBP < 91 mmHg.
	Control: 55		Age range: 51–81		Local anaesthetic: 17 mg 0.76% bupivacaine at L 4–5		
Kelsaka 2006 [32]	O (8 mg): 25	Orthopaedic surgery	ASA: I–II	Double-blinded	Position: lateral	Ondansetron 8 mg before spinal anaesthesia	Reduction in SBP by > 20% from the baseline value.
	Control: 25		Age range: 20–60		Local anaesthetic: 2.5 mL 0.5% hyperbaric bupivacaine at L 3–4		
Marashi 2014 [2]	O (6 mg):70	Urologic, orthopaedic or gynaecologic surgeries	ASA: I–II	Double-blinded	Position: lateral	Ondansetron 6 mg or 12 mg before spinal anaesthesia	Reduction in MAP by > 20% from the baseline value or MAP < 80 mm Hg.
	O (12 mg):70		Age range: 20–50		Local anaesthetic: 15 mg of 0.5% hypertonic bupivacaine at L 3–4 or L 4–5		
	Control: 70						
Rashad 2013 [42]	O (4 mg):20	Caesarean section	ASA: I–II	Investigator-blinded	Position: Sitting	Ondansetron 4 mg before spinal anaesthesia	Reduction in MAP by > 20% from the baseline value.
	Control: 20		Age range: 20–40		Local anaesthetic: 2 mL 0.5% hyperbaric bupivacaine at L 3–4 or L 4–5		
Marciniak 2015 [33]	O (8 mg): 36	Caesarean section	ASA: I–II	Double-blinded	Position: Sitting	Ondansetron 8 mg before spinal anaesthesia	Reduction in SBP by > 20% from the baseline value or SBP < 90 mmHg.
	Control: 34		Age range: NR		Local anaesthetic: 0.5% hypertonic bupivacaine at L 3–4 or L 4–5		
Mendonça 2021 [12]	O (8 mg): 72	Nonobstetric surgery	ASA: I–II	Double-blinded	Position: Sitting	Ondansetron 8 mg before spinal anaesthesia	Reduction in SBP by > 20% from the baseline value or SBP < 90 mmHg.
	Control: 72		Age range: ≥18		Local anaesthetic: hyperbaric bupivacaine (15 mg or more)		
Mohamed 2021 [11]	O (10 mg): 38	Caesarean section	ASA: I–II	Double-blinded	Position: Sitting	Ondansetron 10 mg before spinal anaesthesia	Reduction in MAP by > 20% from the baseline value.
	Control: 38		Age range: NR		Local anaesthetic: 12.5 mL 0.5% isobaric bupivacaine. at L 3–4		
Mohamed 2018 [34]	O (4 mg): 45	NR	ASA: I	Patient-blinded	Position: Sitting	Ondansetron 4 mg before spinal anaesthesia	Reduction in MAP by > 20% from the baseline value or MAP < 70 mm Hg.
	Control: 45		Age range: 18–45		Local anaesthetic: 2.5–3 mL 0.5% hyperbaric bupivacaine at L 3–4		
Mohammadzadeh 2021 [35]	O (4 mg): 127	Caesarean section	ASA: II	Double-blinded	Position: Sitting	Ondansetron 4 mg after spinal anaesthesia	Reduction in BP by > 20% from the baseline value or BP < 100 mm Hg.
	Control: 127		Age range: 18–40		Local anaesthetic: 12.5 mg isobar bupivacaine at L 3–4 or L 4–5		
Nallam 2017 [36]	O (8 mg): 40	Caesarean section	ASA: I–II	Double-blinded	Position: Sitting	Ondansetron 8 mg before spinal anaesthesia	Reduction in BP by > 20% from the baseline value or MAP below 60 mmHg.
	Control: 40		Age range: 22–32		Local anaesthetic: 12.5 mg 0.5% hyperbaric bupivacaine L 3–4 or L 4–5		
Ortiz-Gómez 2014 [37]	O (2 mg):32	Caesarean section	ASA: I	Double-blinded	Position: Sitting	Ondansetron (2, 4 or 8 mg) before spinal anaesthesia	Reduction in SBP by > 25% from the baseline value.
	O (4 mg):32		Age range: 20–45				
	O (8 mg):32				Local anaesthetic: 0.5% hyperbaric bupivacaine L 3–4 or L 4–5		
	Control: 32						
Owczuk 2008 [38]	O (8 mg): 36	NR	ASA: I–II	Double-blinded	Position: Sitting	Ondansetron 8 mg before spinal anaesthesia	SBP < 90 mmHg.
	Control: 35		Age range: 20–70		Local anaesthetic: 4 mL 0.5% hyperbaric bupivacaine L 3–4 or L 4–5		
Owczuk 2015 [39]	O (8 mg): 26	NR	ASA: I–III	Double-blinded	Position: Sitting	Ondansetron 8 mg before spinal anaesthesia	Reduction in SBP by > 20% from the baseline value or SBP < 90 mmHg.
	Control: 27		Age range: >70		Local anaesthetic: 2.5 to 3 mL 0.5% hyperbaric bupivacaine at L 2–3 or L 3–4 or L 4–5		
Pirat 2005 [40]	O (4 mg): 50	Inguinal hernia, cord hydrocele, and pilonidal sinus	ASA: NR	Double-blinded	Position: Sitting	Ondansetron 4 mg before spinal anaesthesia	Reduction in SBP by > 15% from the baseline value.
	Control: 50		Age range: NR		Local anaesthetic: 12.5 mg or 15 mg 0.5% hyperbaric bupivacaine at L 2–3 or L 3–4		
Ramon 2017 [41]	O (8 mg): 65	Caesarean section	ASA: I	Double-blinded	Position: Sitting	Ondansetron 8 mg before spinal anaesthesia	Reduction in SBP by > 25% from the baseline value.
	Control: 65		Age range: 20–45		Local anaesthetic: 0.5% hyperbaric bupivacaine at L 3–4 or L 4–5		
Safavi 2014 [43]	O (8 mg): 40	Orthopaedic surgery	ASA: I–II	Double-blinded	Position: Sitting	Ondansetron 8 mg before spinal anaesthesia	Reduction in SBP by > 20% from the baseline value.
	Control: 40		Age range: 16–65		Local anaesthetic: 0.5% hyperbaric bupivacaine at L 3–4		
Sahoo 2012 [44]	O (4 mg): 26	Caesarean section	ASA: I	Double-blinded	Position: Sitting	Ondansetron 4 mg before spinal anaesthesia	SBP < 90 mmHg or DBP < 60 mmHg.
	Control: 26		Age range: 20–40		Local anaesthetic: 2 mL 0.5% hyperbaric bupivacaine at L 3–4 or L 4–5		
Shakya 2010 [45]	O (4 mg): 40	General and gynaecological surgery	ASA: I	Double-blinded	Position: NR	Ondansetron 4 mg after spinal anaesthesia	NR.
	Control: 40		Age range: NR		Local anaesthetic: 3 mL 0.5% hyperbaric bupivacaine at L 3–4 or L 4–5		
Tatikonda 2019 [46]	O (4 mg): 70	Orthopaedic, gynaecological, and general surgical procedures	ASA: I–II	Double-blinded	Position: Sitting	Ondansetron 4 mg before spinal anaesthesia	Reduction in MAP by > 20% from the baseline value.
	Control: 70		Age range: 20–60		Local anaesthetic: 3 mL 0.5% hyperbaric bupivacaine at L 3–4 or L 4–5		
Terkawi 2015 [47]	O (8 mg): 44	Caesarean section	ASA: I	Double-blinded	Position: recumbent	Ondansetron 8 mg before spinal anaesthesia	SBP < 90 mmHg.
	Control: 42		Age range: NR		Local anaesthetic: 15 mg 0.75% bupivacaine at L 3–4 or L 4–5		
Trabelsi 2015 [48]	O (4 mg): 40	Caesarean section	ASA: NR	Double-blinded	Position: sitting	Ondansetron 4 mg before spinal anaesthesia	Reduction in SBP by > 20% from the baseline value or SBP < 80 mmHg.
	Control: 40		Age range: NR		Local anaesthetic: 2 mL hyperbaric bupivacaine at L 2–3 or L 3–4		
Wang M 2014 [49]	O (2 mg): 29	Caesarean section	ASA: I–II	Double-blinded	Position: NR	Ondansetron (2, 4, 6, or 8 mg) before spinal anaesthesia	Reduction in SBP by > 20% from the baseline value.
	O (4 mg): 30		Age range: 18–35		Local anaesthetic: 2 mL 0.5% hyperbaric bupivacaine		
	O (6 mg): 29						
	O (8 mg): 30						
	Control: 30						
Wang Q 2014 [50]	O (4 mg): 33	Caesarean section	ASA: I–II	Double-blinded	Position: NR	Ondansetron 4 mg before spinal anaesthesia	Reduction in SBP by > 20% from the baseline value.
	Control: 32		Age range: 18–35		Local anaesthetic: 2 mL 0.5% hyperbaric bupivacaine		

Abbreviation: O, ondansetron; NR, not reported; SBP, systolic blood pressure; MAP, mean arterial pressure; BP, blood pressure; DBP, diastolic blood pressure.

**Table 2:** The overall results of the GRADE evaluation.

Outcome	MD/RR (95% CI)	Level of Certainty	Reasons
Incidence of hypotension	0.65 (0.53, 0.80)	⨁⨁⨁◯ MODERATE	Inconsistency was “serious”.
Incidence of bradycardia	0.56 (0.38, 0.83)	⨁⨁⨁⨁ HIGH	None.
Rescue of vasopressor administration	0.50 (0.38, 0.67)	⨁⨁⨁⨁ HIGH	None.
Administration of ephedrine	−2.81 (−4.72, −0.89)	⨁⨁⨁◯ MODERATE	Inconsistency was “serious”.

MD, mean difference; RR, risk ratio; ⨁, not serious; ◯, serious.

## Data Availability

All data in this article are included within the article and Supplementary Materials.

## Supplementary Materials

The following supporting information can be downloaded at: https://www.mdpi.com/article/10.3390/ph15121588/s1, Figure S1: Forest plot of the pooled analysis showing the subgroup analysis for the incidence of hypotension according to different dosage of ondansetron, Figure S2: Forest plot of the pooled analysis showing the subgroup analysis for the incidence of hypotension according to different type of surgical modalities, Figure S3: Sensitivity analysis for the incidence of hypotension.
