# Supplementary material for: Ondansetron Reduces the Incidence of Hypotension after Spinal Anaesthesia: A Systematic Review and Meta-Analysis
Source: Pharmaceuticals (Basel). 2022 Dec 19;15(12):1588. doi: 10.3390/ph15121588 (PMC9787671; doi:10.3390/ph15121588)

Search strategy for Pubmed:

(ondansetron) OR(serotonin antagonist) OR ((“serotonin”[MeSH Terms] OR serotonin[Text Word]) AND antagonist[All Fields]) OR (“Serotonin 5-HT3 Receptor Antagonists”[Mesh]) OR OR (“5-Hydroxytryptamine3”[Mesh])) AND ((“Anesthesia, Spinal”[Mesh]) OR (combined spinal anesthesia) OR ((analgesia OR anesthesia) AND ((spinal OR intrathecal OR subarachnoid)))).

Supplement Figure S1. Forest plot of the pooled analysis showing the subgroup analysis for the incidence of hypotension according to different dosage of ondansetron.

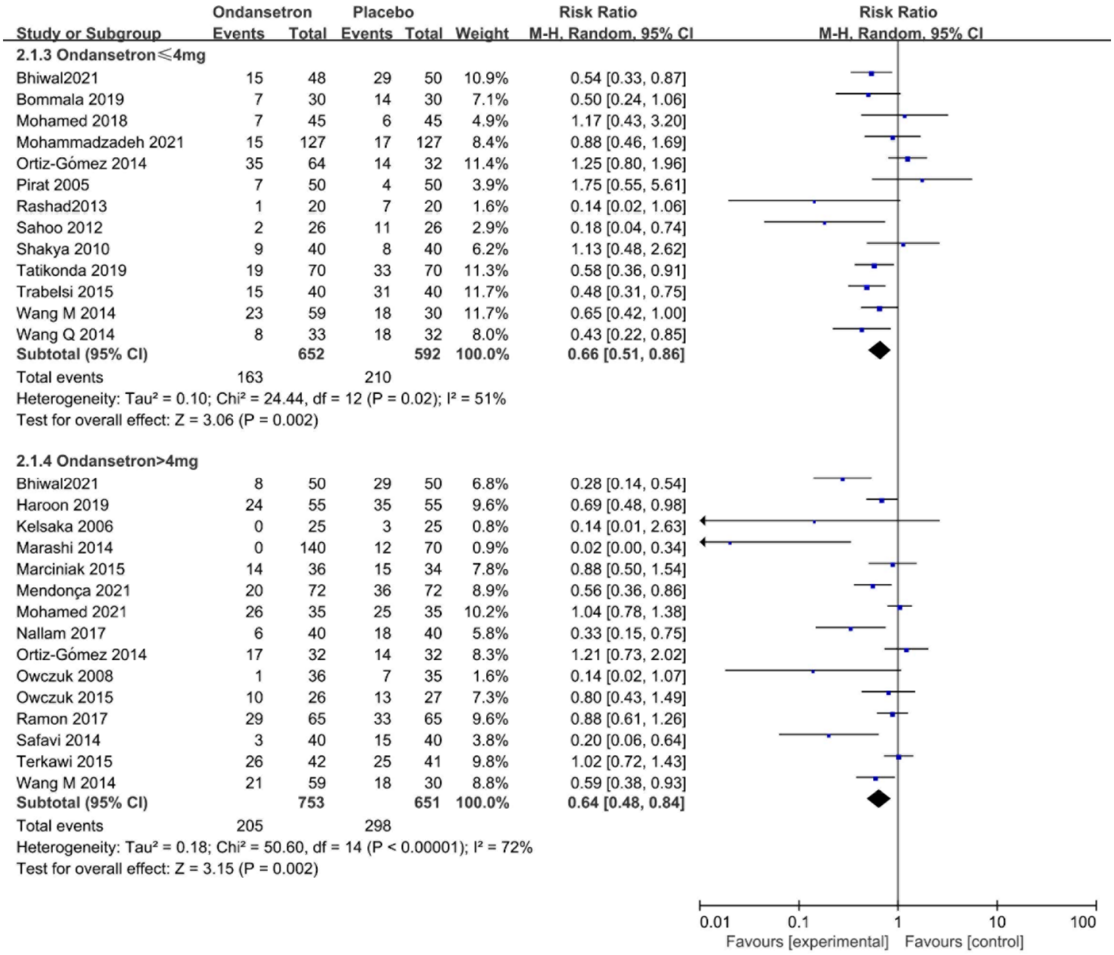

Supplement Figure S2. Forest plot of the pooled analysis showing the subgroup analysis for the incidence of hypotension according to different type of surgical modalities.

Study or Subgroup	Ondansetron Events	Ondansetron Total	Placebo Events	Placebo Total	Weight	Risk Ratio M-H, Random, 95% CI
**2.3.1 Cesarean section**						
Bhiwal2021	23	98	29	50	9.2%	0.40 [0.26, 0.62]
Kelsaka 2006	0	25	3	25	0.8%	0.14 [0.01, 2.63]
Marciniak 2015	14	36	15	34	7.9%	0.88 [0.50, 1.54]
Mohamed 2021	26	35	25	35	10.6%	1.04 [0.78, 1.38]
Mohammadzadeh 2021	15	127	17	127	7.0%	0.88 [0.46, 1.69]
Nallam 2017	6	40	18	40	5.6%	0.33 [0.15, 0.75]
Ortiz-Gómez 2014	52	96	14	32	9.1%	1.24 [0.80, 1.91]
Ramon 2017	29	65	33	65	9.9%	0.88 [0.61, 1.26]
Rashad2013	1	20	7	20	1.5%	0.14 [0.02, 1.06]
Sahoo 2012	2	26	11	26	2.7%	0.18 [0.04, 0.74]
Terkawi 2015	26	42	25	41	10.1%	1.02 [0.72, 1.43]
Trabelsi 2015	15	40	31	40	9.1%	0.48 [0.31, 0.75]
Wang M 2014	44	118	18	30	9.8%	0.62 [0.43, 0.90]
Wang Q 2014	8	33	18	32	6.7%	0.43 [0.22, 0.85]
**Subtotal (95% CI)**		**801**		**597**	**100.0%**	**0.66 [0.51, 0.86]**
Total events	261		264			
Heterogeneity: Tau² = 0.15; Chi² = 43.74, df = 13 (P < 0.0001); I² = 70%						
Test for overall effect: Z = 3.07 (P = 0.002)						
**2.3.2 Non-cesarean section**						
Bommala 2019	7	30	14	30	10.8%	0.50 [0.24, 1.06]
Haroon 2019	24	55	35	55	19.1%	0.69 [0.48, 0.98]
Marashi 2014	0	140	12	70	1.3%	0.02 [0.00, 0.34]
Mendonça 2021	20	72	36	72	17.2%	0.56 [0.36, 0.86]
Owczuk 2015	10	26	13	27	13.1%	0.80 [0.43, 1.49]
Pirat 2005	7	50	4	50	6.1%	1.75 [0.55, 5.61]
Safavi 2014	3	40	15	40	6.1%	0.20 [0.06, 0.64]
Shakya 2010	9	40	8	40	9.4%	1.13 [0.48, 2.62]
Tatikonda 2019	19	70	33	70	16.8%	0.58 [0.36, 0.91]
**Subtotal (95% CI)**		**523**		**454**	**100.0%**	**0.62 [0.45, 0.87]**
Total events	99		170			
Heterogeneity: Tau² = 0.11; Chi² = 16.85, df = 8 (P = 0.03); I² = 53%						
Test for overall effect: Z = 2.82 (P = 0.005)						

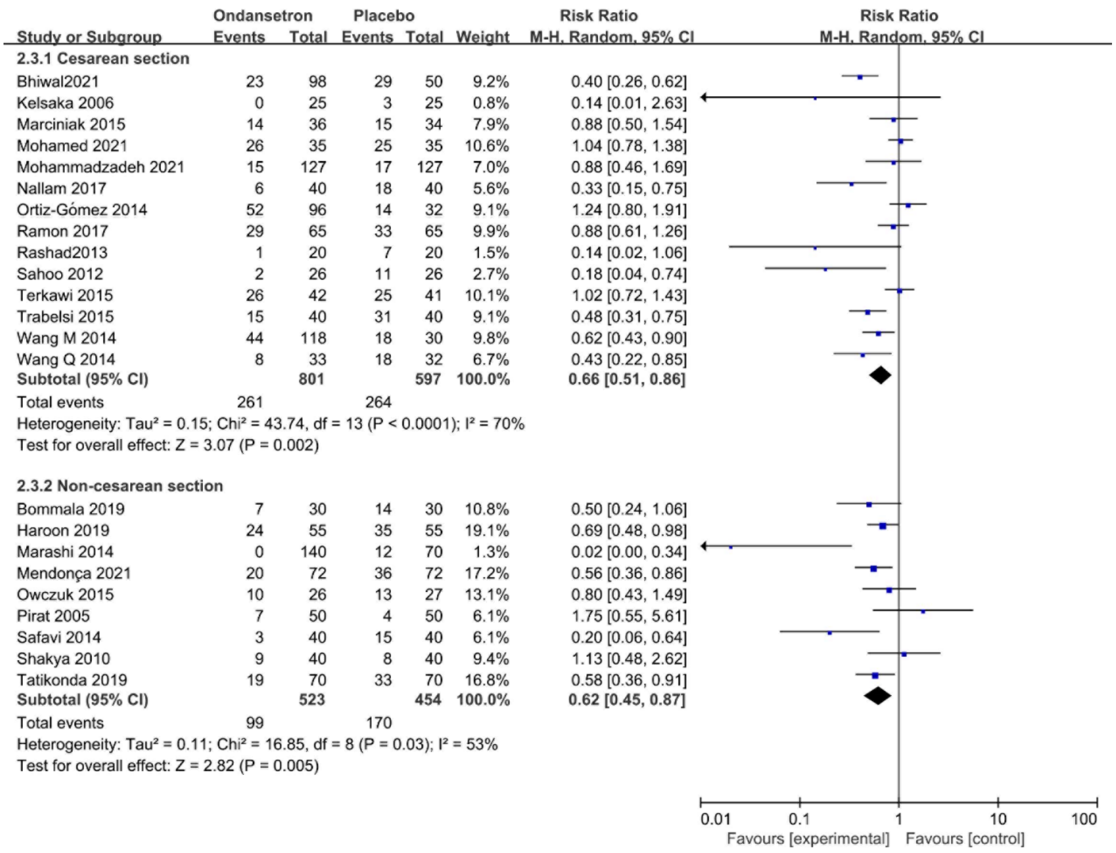

Supplement Figure S3. Sensitivity analysis for the incidence of hypotension.

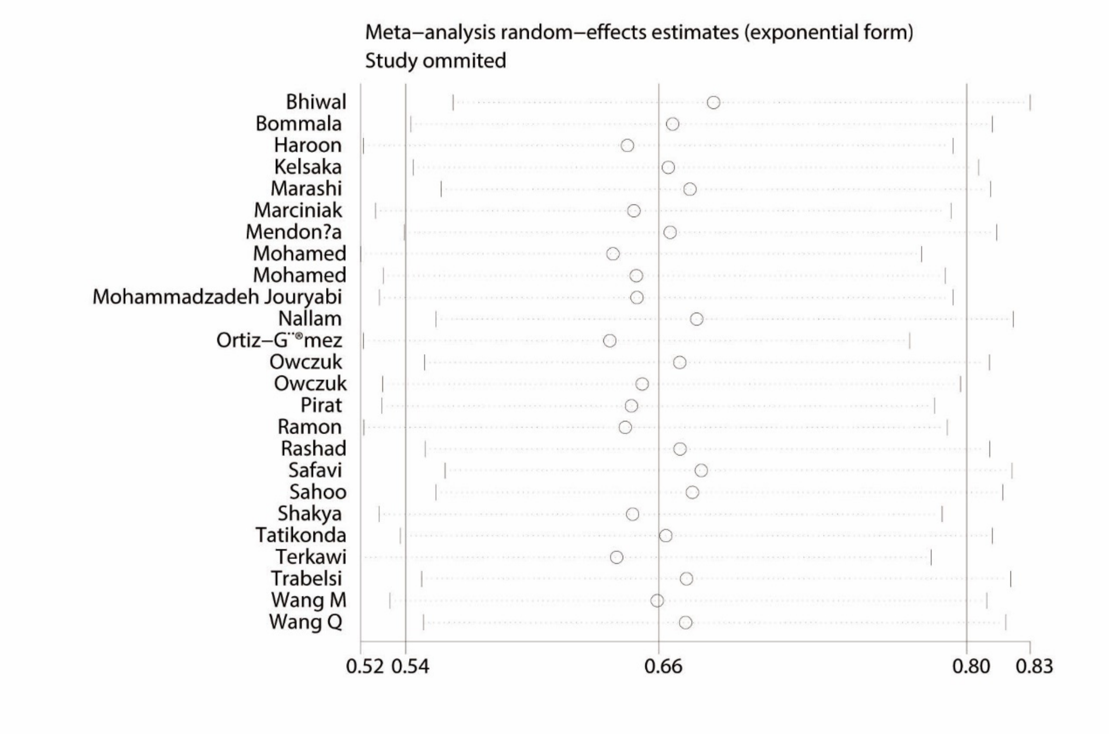

Supplement: Supplementary file 1 [file pharmaceuticals-15-01588-s001.zip › pharmaceuticals-2006873-supplementary.pdf]
